# Editorial: Biodegradation of High Molecular Weight Polyaromatic Hydrocarbons in Different Environments

**DOI:** 10.3389/fmicb.2021.704897

**Published:** 2021-07-22

**Authors:** Piyush Pandey, Atya Kapley, Satinder Kaur Brar

**Affiliations:** ^1^Soil and Environmental Microbiology Lab, Department of Microbiology, Assam University, Silchar, India; ^2^Environmental Biotechnology and Genomics, National Environmental Engineering Research Institute, Nagpur, India; ^3^Department of Civil Engineering, Suite 337 Bergeron Centre for Engineering Excellence, Toronto, ON, Canada

**Keywords:** polyaromatic hydrocarbon, bioremediaiton, rhizoremediation, biostimulation and bioaugmentation, hydrocarbon contaminants

Polyaromatic hydrocarbons (PAHs) are hazardous organic molecules with mutagenic, carcinogenic and genotoxic effects. According to the United States Environmental Protection Agency (USEPA) total 16 PAHs are considered as priority pollutants and are toxic for living organisms (Mrozik et al., 2003). The worldwide use and distribution of PAHs leads to their entry into the environment and bioaccumulation in different food chains (Kotoky et al., 2018). Due to their persistent nature and high hydrophobicity, PAHs molecules bind themselves to soil particles making them unavailable for biodegradation (Nanca et al., 2018). The Exxon Valdez oil spill in 1989 initiated a worldwide fervor in the research on PAH degradation and three decades later, novel strategies and new tools are adding new dimensions in tackling this problem.

Though several methods have been suggested for the removal of PAHs, including incineration, oxidation, and fixation, what is often ignored is the importance of improving their bio-availability. Conventional methods sometimes do not remove PAHs completely, but instead, produce fractional products which are more toxic than the parent molecules (Gan et al., 2009). Bioremediation is considered the most efficient, cost-effective eco-friendly technique for the removal of PAHs (Figure 1). Bacteria harbor different mechanisms to degrade PAHs and other xenobiotic compounds (Kotoky et al., 2018). *Pseudomonas aeruginosa, P. fluoresens, Mycobacterium* spp*., Haemophilus* spp*., Rhodococcus* spp*., Paenibacillus* spp. are some of the commonly studied PAHs-degrading bacteria (Lu et al., 2019). Biosurfactant producing microbes are a very important community to make the contaminants available for biodegradation (Gan et al., 2009; Singha and Pandey, 2021).

**Figure 1:**
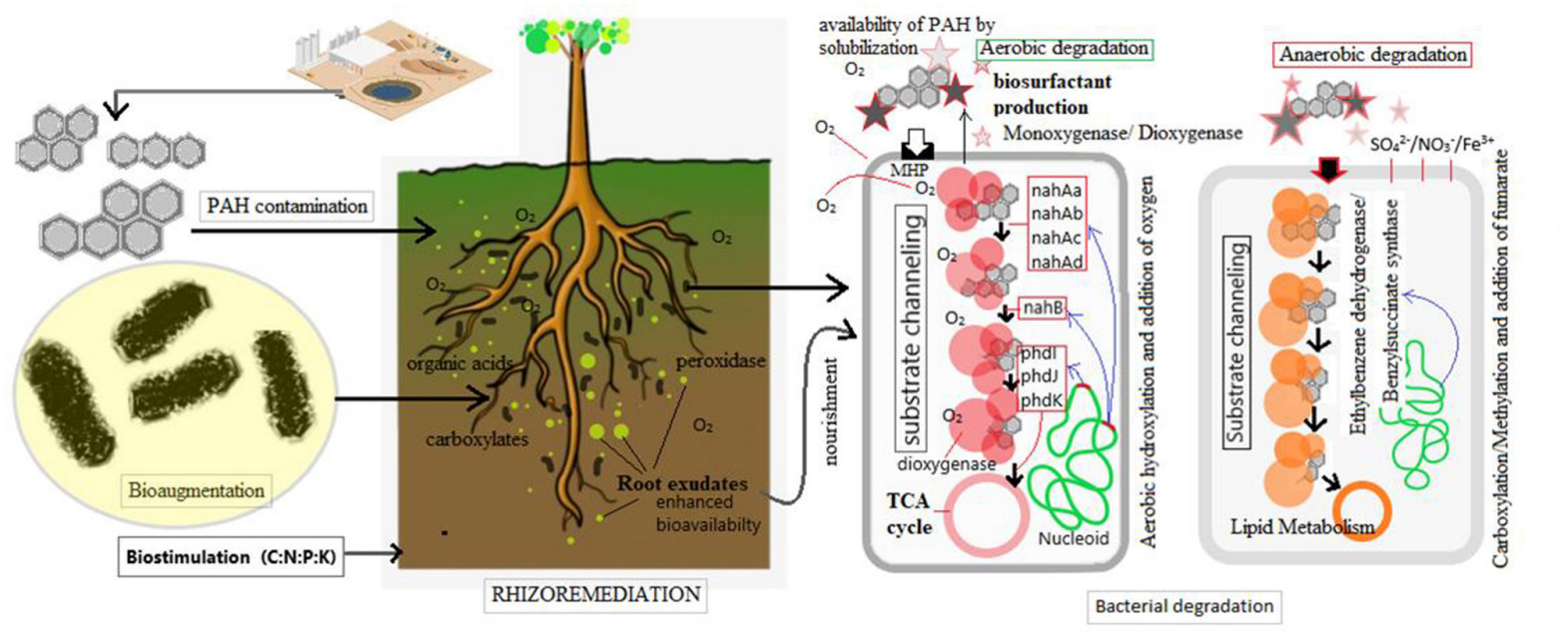
Different approaches for the bioremediation of Polyaromatic Hydrocarbon contamianted environments.

Recent advancement in genomics, proteomics and transcriptomics allow a more enriched knowledge in PAH degradation and provide insights of the different metabolic pathways involved. Real-time PCR, DNA-based stable isotope probing, single-cell genomics and DNA microarray techniques are being used in contaminated areas in order to monitor the PAH catabolic gene expression. Since PAHs have complex degradative pathways, genome sequencing helps decipher the potential catabolic activity and enables the knowledge-based designing of a consortia (Kotoky and Pandey, 2020, 2021). High-throughput Illumina sequencing has been extensively used for the detection of unculturable microorganisms involved in PAH degradation (Wang et al., 2018). Two techniques, metaproteomics and metabolomics, have been very useful in the identification of proteins and metabolites produced during the biodegradation process.

Land farming has been recommended for large-scale application, in which, the indigenous microbial population is stimulated for biodegradation of PAHs. Other techniques such as bioaugmentation, composting, biopiling, phytoremediation, vermiremediation, using plant growth promoting bacteria (PGPB) in contaminated sites have also emerged to be highly effective and provide a considerable amount of PAH removal (Vázquez-Núñez et al., 2020). This issue addresses this concern with selected reviews and research articles from leading researchers.

In this issue, in one of the articles, the microbial electrochemical system (MES) of specific designs for PAH removal has been elaborated with unique details (Hao et al.). In this technique, a solid anode is utilized as an inexhaustible electron acceptor and microbial activity is enriched by biocurrent *in situ* to confirm removal of PAHs. The review elaborates the degradation of PAHs using MES, as well as its effectiveness in different settings, such as modification of the anode, improvement of substrate and transfer of an electron, supplementation of chemical reagents. The mass transfer of PAHs combined with phytoremediation, functional microorganisms and exoelectrogens, electrochemical activity and internal resistance, the power density and current density of the PAH removal system have also been explained (Hao et al.). Further, an overview of physical and chemical techniques for the remediation of PAHs such as adsorption, soil filtration, membrane filtration, thermal-, or electrokinetic- oxidation and photocatalytic treatments has been discussed. In addition, a detailed systematic compilation of the use of microbial treatments, *in-situ* and *ex-situ* biological treatments like bioaugmentation, land-farming, biostimulation, phytoremediation, bioreactor, vermiremediation and generation of value-added by-products during degradation of PAHs has been elaborated, which will be highly useful for scale-up of technologies at commercial level (Patel et al.). Rhizoremediation is one of the strategies in bioremediation that uses plant-microbe interaction for the removal of higher-molecular-weight PAHs (Singha and Pandey, 2021). An ornamental plant, *Tagetes erecta* L. has been reported to be an efficient rhizoremediator of pyrene, when applied with a non-pathogenic isolate of *Klebsiella pneumoniae*. Also, this interaction induced a shift in the microbial community in favor of degradation of xenobiotic compounds when supplemented with the bacterial isolate, as assessed through metagenomic analysis of pyrene contaminated soil (Rajkumari et al.)

One of the main sources of PAH contamination is the use of crude oil. The process by which crude oil is sourced and available for use is known as the oil supply chain during which crude oil is subjected to acidification, bio-deterioration, which affects the environment and economy. Genes and pathways involved in PAH degradation in different stages of the oil supply chain had been described that adversely affect various environments (seawater, groundwater, oil reservoir) and present challenges for PAH bioremediation for oil spills (Hidalgo et al.)

Not only bacteria but fungi have also been exploited for PAH removal from contaminated environments. The role of fungi in the long-term natural bioremediation of contaminated soils has been assessed, and reported, and summarized as it could also contribute to the development of a fungal community adapted to the contaminated soils. Next-generation sequencing is accompanied with the microbiome and functional diversity of fungi based on Biolog FFPlates, glomalin-related soil protein (GRSP) content, trace element and PAH concentration. In this study, soils collected from oil wells had shown to be more diversified with higher biological activity (35% share the PAHs degrading population) as compared with 3 m radius of the oil wells (<5% share the PAHs degrading population) (Gałazka et al.) In another paper, the potential of fungal bioaugmentation was utilized as an efficient strategy to improve the bioremediation of an aged industrially polluted soil enriched with heavy hydrocarbon. A consortium of six potentially hydrocarbonoclastic fungi was applied, and compared with soil biostimulation process (water and nutrient addition). Bioaugmentation of indigenous fungi resulted in an important shift of the bacterial populations, which was also linked to HMW-PAHs biodegradation efficiency, compared to biostimulation. Also, the fungal bioaugmentation with indigenous fungi exerted an influence on the soil bacterial populations toward a more diverse microbial community (Medaura et al.)

Innovative strategies are being devised for removal of HMW-PAHs from the contaminated environments. Some of the recent and promising technologies have been described in this issue. However, there had been concerns for the efficiency of each such technology (Gan et al., 2009; Singha and Pandey, 2021). In fact, the HMW-PAHs are often present in the environment with several other persistent co-contaminants, like heavy metals and other hydrocarbons. Therefore, it is relevant to suggest that the technologies should be developed with multi-dimensional approach, where more than one, technically-feasible technologies, should be applied together to achieve maximum efficiency of HMW-PAHs removal from contaminated environments.

## Author Contributions

PP prepared the draft and finalized the manuscript. AK and SB edited and corrected the draft. All authors contributed to the article and approved the submitted version.

## Conflict of Interest

The authors declare that the research was conducted in the absence of any commercial or financial relationships that could be construed as a potential conflict of interest.
